# Supplementary material for: The Prevalence of Dysphagia in Individuals Living in Residential Aged Care Facilities: A Systematic Review and Meta-Analysis
Source: Healthcare (Basel). 2024 Mar 13;12(6):649. doi: 10.3390/healthcare12060649 (PMC10970675; doi:10.3390/healthcare12060649)
Supplement: Supplementary file 1 [file healthcare-12-00649-s001.zip › healthcare-2865736-supplementary.pdf]

Supplementary Table S1. Search terms.

Prevalence	Prevalence OR prevalen* OR frequen* OR occur* OR risk OR incidence OR inciden*
Dysphagia	Dysphagia OR “deglutition disorders” OR “deglutition disorder*” OR swallow* OR deglutition
Assessment	“Instrumental assessment” OR “swallow* evaluation” OR “swallow* assessment” OR “swallow* exam” OR “Speech pathologist” OR “Speech and language pathologist” OR “Modified barium swallow” OR MBS OR “Video-fluoroscopic swallowing study” OR VFSS OR “Videofluoroscopic swallowing study” OR “Fibreoptic Endoscopic Evaluation of Swallowing” OR “Flexible endoscopic Evaluation of Swallowing” OR FEES OR “High resolution manometry” OR Ultrasound OR “Speech-Language pathology” OR Ultrasonography
Residential aged care	“Homes for the aged” OR “home* for the age*” OR “Nursing homes” OR “Nursing home*”OR “Long term Care” OR “Skilled nursing facility” OR “Skilled Nursing facilit*” OR “Residential facilities” OR “Residential facilit*” OR “Geriatric institution*” OR “Assisted living” OR “aged care” OR “housing for the elderly” OR “residential care” OR “care home*” OR “retirement” OR “elderly care” OR “geriatric care”
Nervous system diseases	“Nervous system diseases” OR “Nervous system disease*” OR “Neurologic* disorder*” OR “Neurologic* disease*” OR “Neurodegenerative diseases” OR “Neurodegenerative disease*” OR “Neurodegenerative disorder*”
Dementia	Dementia

Malnutrition	Malnutrition OR "Protein-energy malnutrition" OR "Weight loss" OR "Nutritional status" OR Malnourished OR Malnourishment OR Malnourish* OR Undernutrition OR Undernourished OR "Risk of malnutrition" OR "subjective global assessment" OR SGA OR "patient generated subjective global assessment" OR "PG-SGA" OR "mini nutritional assessment" OR MNA OR "mini nutritional assessment short form" OR MNA-SF OR "nutrition assessment" OR "geriatric assessment" OR "malnutrition screening"
Dentition	Dentition OR Dentures OR Denture* OR Dental OR Oral OR Teeth

Supplementary Table S2. Search Strategies as per database.

Scopus

S1
TITLE – ABS – KEY (Dysphagia OR “Deglutition disorders” OR “Deglutition disorder*” OR swallow* OR deglutition) OR TITLE – ABS – KEY (“Instrumental assessment” OR “Swallow* evaluation” OR “Swallow* assessment” OR “Swallow* exam” OR “Speech pathologist” OR “Speech and language pathologist” OR “Modified barium swallow” OR MBS OR “Video-fluoroscopic swallowing study” OR VFSS OR “Videofluoroscopic swallowing study” OR “Fibreoptic Endoscopic Evaluation of Swallowing” OR “Flexible endoscopic Evaluation of Swallowing” OR FEES OR “High resolution manometry” OR Ultrasound OR “Speech-Language pathology” OR Ultrasonography)

S2
TITLE – ABS – KEY (Prevalence OR Prevalen* OR Frequen* OR Occur* OR Risk OR Incidence OR Inciden*) AND TITLE – ABS – KEY (“Homes for the aged” OR “Home* for the age*” OR “Nursing homes” OR “Nursing home*”OR “Long term Care” OR “Skilled nursing facility” OR “Skilled Nursing facilit*” OR “Residential facilities” OR “Residential facilit*” OR “Geriatric institution*” OR “Assisted living” OR “aged care” OR “Housing for the elderly” OR “residential care” OR “care home*” OR “retirement” OR “elderly care” OR “Geriatric care”)

S3
S1 AND S2

S4
TITLE – ABS – KEY (“Nervous system diseases” OR “Nervous system disease*” OR “Neurologic* disorder*” OR “Neurologic* disease*” OR “Neurodegenerative diseases” OR “Neurodegenerative disease*” OR “Neurodegenerative disorder*”)

S5
S3 AND S4

S6
TITLE – ABS – KEY “Dementia”

S7
S3 and S6

S8
TITLE – ABS – KEY (Malnutrition OR “Protein-energy malnutrition” OR “Weight loss” OR “Nutritional status” OR Malnourished OR Malnourishment OR Malnourish* OR Undernutrition OR Undernourished OR “Risk of malnutrition” OR “subjective global

assessment” OR SGA OR “patient generated subjective global assessment” OR “PG-SGA” OR “mini nutritional assessment” OR MNA OR “mini nutritional assessment short form” OR MNA-SF OR “nutrition assessment” OR “geriatric assessment” OR “malnutrition screening”)<br><br>S9<br>S3 AND S8<br><br>S10<br>TITLE – ABS – KEY (“Dentition” OR Dentures OR “Denture*”OR “Dental” OR “Oral” OR “Teeth”)<br><br>S11<br>S3 AND S10<br><br>S12 – Together – Search with OR:<br>S3 OR S5 OR S7 OR S9 OR S11<br>*Results: 3,693*
**Web of science – Core collection**<br><br>S1<br>TITLE – ABS – KEY (Dysphagia OR “Deglutition disorders” OR “Deglutition disorder*” OR swallow* OR deglutition) OR TITLE – ABS – KEY (“Instrumental assessment” OR “Swallow* evaluation” OR “Swallow* assessment” OR “Swallow* exam” OR “Speech pathologist” OR “Speech and language pathologist” OR “Modified barium swallow” OR MBS OR “Video-fluoroscopic swallowing study” OR VFSS OR “Videofluoroscopic swallowing study” OR “Fibreoptic Endoscopic Evaluation of Swallowing” OR “Flexible endoscopic Evaluation of Swallowing” OR FEES OR “High resolution manometry” OR Ultrasound OR “Speech-Language pathology” OR Ultrasonography)<br><br>S2<br>TITLE – ABS – KEY (Prevalence OR Prevalen* OR Frequen* OR Occur* OR Risk OR Incidence OR Inciden*) AND TITLE – ABS – KEY (“Homes for the aged” OR “Home* for the age*” OR “Nursing homes” OR “Nursing home*”OR “Long term Care” OR “Skilled nursing facility” OR “Skilled Nursing facilit*” OR “Residential facilities” OR “Residential facilit*” OR “Geriatric institution*” OR “Assisted living” OR “aged care” OR “Housing for the elderly” OR “residential care” OR “care home*” OR “retirement” OR “elderly care” OR “Geriatric care”)<br><br>S3<br>S1 AND S2<br><br>S4<br>TITLE – ABS – KEY (“Nervous system diseases” OR “Nervous system disease*” OR “Neurologic* disorder*” OR “Neurologic* disease*” OR “Neurodegenerative diseases” OR “Neurodegenerative disease*” OR “Neurodegenerative disorder*”)<br><br>S5<br>S3 AND S4

S6
TITLE – ABS – KEY “Dementia”

S7
S3 and S6

S8
TITLE – ABS – KEY (Malnutrition OR “Protein-energy malnutrition” OR “Weight loss” OR “Nutritional status” OR Malnourished OR Malnourishment OR Malnourish* OR Undernutrition OR Undernourished OR “Risk of malnutrition” OR “subjective global assessment” OR SGA OR “patient generated subjective global assessment” OR “PG-SGA” OR “mini nutritional assessment” OR MNA OR “mini nutritional assessment short form” OR MNA-SF OR “nutrition assessment” OR “geriatric assessment” OR “malnutrition screening”)

S9
S3 AND S8

S10
TITLE – ABS – KEY (“Dentition” OR Dentures OR “Denture*”OR “Dental” OR “Oral” OR “Teeth”)

S11
S3 AND S10

S12 – Together – Search with OR:
S3 OR S5 OR S7 OR S9 OR S11
Results: 1,064

MEDLINE

S1
MH (Dysphagia OR “Deglutition disorders” OR “Deglutition disorder*” OR swallow* OR deglutition) OR TI (Dysphagia OR “Deglutition disorders” OR “Deglutition disorder*” OR swallow* OR deglutition) OR AB (Dysphagia OR “Deglutition disorders” OR “Deglutition disorder*” OR swallow* OR deglutition)

S2
MH (“Instrumental assessment” OR “Swallow* evaluation” OR “Swallow* assessment” OR “Swallow* exam” OR “Speech pathologist” OR “Speech and language pathologist” OR “Modified barium swallow” OR MBS OR “Video-fluoroscopic swallowing study” OR VFSS OR “Videofluoroscopic swallowing study” OR “Fibreoptic Endoscopic Evaluation of Swallowing” OR “Flexible endoscopic Evaluation of Swallowing” OR FEES OR “High resolution manometry” OR Ultrasound OR “Speech-Language pathology” OR Ultrasonography) OR TI (“Instrumental assessment” OR “Swallow* evaluation” OR “Swallow* assessment” OR “Swallow* exam” OR “Speech pathologist” OR “Speech and language pathologist” OR “Modified barium swallow” OR MBS OR “Video-fluoroscopic swallowing study” OR VFSS OR “Videofluoroscopic swallowing study” OR “Fibreoptic Endoscopic Evaluation of Swallowing” OR “Flexible endoscopic Evaluation of Swallowing” OR FEES OR “High resolution manometry” OR Ultrasound OR “Speech-Language

pathology” OR Ultrasonography) OR AB (“Instrumental assessment” OR “Swallow* evaluation” OR “Swallow* assessment” OR “Swallow* exam” OR “Speech pathologist” OR “Speech and language pathologist” OR “Modified barium swallow” OR MBS OR “Video-fluoroscopic swallowing study” OR VFSS OR “Videofluoroscopic swallowing study” OR “Fibreoptic Endoscopic Evaluation of Swallowing” OR “Flexible endoscopic Evaluation of Swallowing” OR FEES OR “High resolution manometry” OR Ultrasound OR “Speech-Language pathology” OR Ultrasonography)

S3
S1 OR S2

S4
MH (Prevalence OR Prevalen* OR Frequen* OR Occur* OR Risk OR Incidence OR Inciden*) OR TI (Prevalence OR Prevalen* OR Frequen* OR Occur* OR Risk OR Incidence OR Inciden*) OR AB (Prevalence OR Prevalen* OR Frequen* OR Occur* OR Risk OR Incidence OR Inciden*)

S5
MH (“Homes for the aged” OR “Home* for the age*” OR “Nursing homes” OR “Nursing home*”OR “Long term Care” OR “Skilled nursing facility” OR “Skilled Nursing facilit*” OR “Residential facilities” OR “Residential facilit*” OR “Geriatric institution*” OR “Assisted living” OR “aged care” OR “Housing for the elderly” OR “residential care” OR “care home*” OR “retirement” OR “elderly care” OR “Geriatric care”) OR TI (“Homes for the aged” OR “Home* for the age*” OR “Nursing homes” OR “Nursing home*”OR “Long term Care” OR “Skilled nursing facility” OR “Skilled Nursing facilit*” OR “Residential facilities” OR “Residential facilit*” OR “Geriatric institution*” OR “Assisted living” OR “aged care” OR “Housing for the elderly” OR “residential care” OR “care home*” OR “retirement” OR “elderly care” OR “Geriatric care”) OR AB (“Homes for the aged” OR “Home* for the age*” OR “Nursing homes” OR “Nursing home*”OR “Long term Care” OR “Skilled nursing facility” OR “Skilled Nursing facilit*” OR “Residential facilities” OR “Residential facilit*” OR “Geriatric institution*” OR “Assisted living” OR “aged care” OR “Housing for the elderly” OR “residential care” OR “care home*” OR “retirement” OR “elderly care” OR “Geriatric care”)

S6
S3 AND S4 AND S5

S7
MH (“Nervous system diseases” OR “Nervous system disease*” OR “Neurologic* disorder*” OR “Neurologic* disease*” OR “Neurodegenerative diseases” OR “Neurodegenerative disease*” OR “Neurodegenerative disorder*”) OR TI (“Nervous system diseases” OR “Nervous system disease*” OR “Neurologic* disorder*” OR “Neurologic* disease*” OR “Neurodegenerative diseases” OR “Neurodegenerative disease*” OR “Neurodegenerative disorder*”) OR AB (“Nervous system diseases” OR “Nervous system disease*” OR “Neurologic* disorder*” OR “Neurologic* disease*” OR “Neurodegenerative diseases” OR “Neurodegenerative disease*” OR “Neurodegenerative disorder*”)

S8
S6 AND S7

S9<br>MH (Dementia) OR TI (Dementia) OR AB (Dementia)<br><br>S10<br>S6 AND S9<br><br>S11<br>MH (Malnutrition OR “Protein-energy malnutrition” OR “Weight loss” OR “Nutritional status” OR Malnourished OR Malnourishment OR Malnourish* OR Undernutrition OR Undernourished OR “Risk of malnutrition” OR “subjective global assessment” OR SGA OR “patient generated subjective global assessment” OR “PG-SGA” OR “mini nutritional assessment” OR MNA OR “mini nutritional assessment short form” OR MNA-SF OR “nutrition assessment” OR “geriatric assessment” OR “malnutrition screening”) OR TI (Malnutrition OR “Protein-energy malnutrition” OR “Weight loss” OR “Nutritional status” OR Malnourished OR Malnourishment OR Malnourish* OR Undernutrition OR Undernourished OR “Risk of malnutrition” OR “subjective global assessment” OR SGA OR “patient generated subjective global assessment” OR “PG-SGA” OR “mini nutritional assessment” OR MNA OR “mini nutritional assessment short form” OR MNA-SF OR “nutrition assessment” OR “geriatric assessment” OR “malnutrition screening”) OR AB (Malnutrition OR “Protein-energy malnutrition” OR “Weight loss” OR “Nutritional status” OR Malnourished OR Malnourishment OR Malnourish* OR Undernutrition OR Undernourished OR “Risk of malnutrition” OR “subjective global assessment” OR SGA OR “patient generated subjective global assessment” OR “PG-SGA” OR “mini nutritional assessment” OR MNA OR “mini nutritional assessment short form” OR MNA-SF OR “nutrition assessment” OR “geriatric assessment” OR “malnutrition screening”)<br><br>S12<br>S6 AND S11<br><br>S13<br>MH (“Dentition” OR Dentures OR “Denture*”OR “Dental” OR “Oral” OR “Teeth”) OR TI (“Dentition” OR Dentures OR “Denture*”OR “Dental” OR “Oral” OR “Teeth”) OR AB (“Dentition” OR Dentures OR “Denture*” OR “Dental” OR “Oral” OR “Teeth”)<br><br>S14<br>S6 AND S13<br><br>S15 – Together – search with OR:<br>S6 OR S8 OR S10 OR S12 OR S14<br>*Results: 972*
**CINAHL Plus with full text**<br><br>S1<br>MH (Dysphagia OR “Deglutition disorders” OR “Deglutition disorder*” OR swallow* OR deglutition) OR TI (Dysphagia OR “Deglutition disorders” OR “Deglutition disorder*” OR swallow* OR deglutition) OR AB (Dysphagia OR “Deglutition disorders” OR “Deglutition disorder*” OR swallow* OR deglutition)<br><br><br>S2

MH (“Instrumental assessment” OR “Swallow* evaluation” OR “Swallow* assessment” OR “Swallow* exam” OR “Speech pathologist” OR “Speech and language pathologist” OR “Modified barium swallow” OR MBS OR “Video-fluoroscopic swallowing study” OR VFSS OR “Videofluoroscopic swallowing study” OR “Fibreoptic Endoscopic Evaluation of Swallowing” OR “Flexible endoscopic Evaluation of Swallowing” OR FEES OR “High resolution manometry” OR Ultrasound OR “Speech-Language pathology” OR Ultrasonography) OR TI (“Instrumental assessment” OR “Swallow* evaluation” OR “Swallow* assessment” OR “Swallow* exam” OR “Speech pathologist” OR “Speech and language pathologist” OR “Modified barium swallow” OR MBS OR “Video-fluoroscopic swallowing study” OR VFSS OR “Videofluoroscopic swallowing study” OR “Fibreoptic Endoscopic Evaluation of Swallowing” OR “Flexible endoscopic Evaluation of Swallowing” OR FEES OR “High resolution manometry” OR Ultrasound OR “Speech-Language pathology” OR Ultrasonography) OR AB (“Instrumental assessment” OR “Swallow* evaluation” OR “Swallow* assessment” OR “Swallow* exam” OR “Speech pathologist” OR “Speech and language pathologist” OR “Modified barium swallow” OR MBS OR “Video-fluoroscopic swallowing study” OR VFSS OR “Videofluoroscopic swallowing study” OR “Fibreoptic Endoscopic Evaluation of Swallowing” OR “Flexible endoscopic Evaluation of Swallowing” OR FEES OR “High resolution manometry” OR Ultrasound OR “Speech-Language pathology” OR Ultrasonography)

S3
S1 OR S2

S4
MH (Prevalence OR Prevalen* OR Frequen* OR Occur* OR Risk OR Incidence OR Inciden*) OR TI (Prevalence OR Prevalen* OR Frequen* OR Occur* OR Risk OR Incidence OR Inciden*) OR AB (Prevalence OR Prevalen* OR Frequen* OR Occur* OR Risk OR Incidence OR Inciden*)

S5
MH (“Homes for the aged” OR “Home* for the age*” OR “Nursing homes” OR “Nursing home*”OR “Long term Care” OR “Skilled nursing facility” OR “Skilled Nursing facilit*” OR “Residential facilities” OR “Residential facilit*” OR “Geriatric institution*” OR “Assisted living” OR “aged care” OR “Housing for the elderly” OR “residential care” OR “care home*” OR “retirement” OR “elderly care” OR “Geriatric care”) OR TI (“Homes for the aged” OR “Home* for the age*” OR “Nursing homes” OR “Nursing home*”OR “Long term Care” OR “Skilled nursing facility” OR “Skilled Nursing facilit*” OR “Residential facilities” OR “Residential facilit*” OR “Geriatric institution*” OR “Assisted living” OR “aged care” OR “Housing for the elderly” OR “residential care” OR “care home*” OR “retirement” OR “elderly care” OR “Geriatric care”) OR AB (“Homes for the aged” OR “Home* for the age*” OR “Nursing homes” OR “Nursing home*”OR “Long term Care” OR “Skilled nursing facility” OR “Skilled Nursing facilit*” OR “Residential facilities” OR “Residential facilit*” OR “Geriatric institution*” OR “Assisted living” OR “aged care” OR “Housing for the elderly” OR “residential care” OR “care home*” OR “retirement” OR “elderly care” OR “Geriatric care”)

S6
S3 AND S4 AND S5

S7

MH (“Nervous system diseases” OR “Nervous system disease*” OR “Neurologic* disorder*” OR “Neurologic* disease*” OR “Neurodegenerative diseases” OR “Neurodegenerative disease*” OR “Neurodegenerative disorder*”) OR TI (“Nervous system diseases” OR “Nervous system disease*” OR “Neurologic* disorder*” OR “Neurologic*

disease*” OR “Neurodegenerative diseases” OR “Neurodegenerative disease*” OR “Neurodegenerative disorder*”) OR AB (“Nervous system diseases” OR “Nervous system disease*” OR “Neurologic* disorder*” OR “Neurologic* disease*” OR “Neurodegenerative diseases” OR “Neurodegenerative disease*” OR “Neurodegenerative disorder*”)

S8
S6 AND S7

S9
MH (Dementia) OR TI (Dementia) OR AB (Dementia)

S10
S6 AND S9

S11
MH (Malnutrition OR “Protein-energy malnutrition” OR “Weight loss” OR “Nutritional status” OR Malnourished OR Malnourishment OR Malnourish* OR Undernutrition OR Undernourished OR “Risk of malnutrition” OR “subjective global assessment” OR SGA OR “patient generated subjective global assessment” OR “PG-SGA” OR “mini nutritional assessment” OR MNA OR “mini nutritional assessment short form” OR MNA-SF OR “nutrition assessment” OR “geriatric assessment” OR “malnutrition screening”) OR TI (Malnutrition OR “Protein-energy malnutrition” OR “Weight loss” OR “Nutritional status” OR Malnourished OR Malnourishment OR Malnourish* OR Undernutrition OR Undernourished OR “Risk of malnutrition” OR “subjective global assessment” OR SGA OR “patient generated subjective global assessment” OR “PG-SGA” OR “mini nutritional assessment” OR MNA OR “mini nutritional assessment short form” OR MNA-SF OR “nutrition assessment” OR “geriatric assessment” OR “malnutrition screening”) OR AB (Malnutrition OR “Protein-energy malnutrition” OR “Weight loss” OR “Nutritional status” OR Malnourished OR Malnourishment OR Malnourish* OR Undernutrition OR Undernourished OR “Risk of malnutrition” OR “subjective global assessment” OR SGA OR “patient generated subjective global assessment” OR “PG-SGA” OR “mini nutritional assessment” OR MNA OR “mini nutritional assessment short form” OR MNA-SF OR “nutrition assessment” OR “geriatric assessment” OR “malnutrition screening”)

S12
S6 AND S11

S13
MH (“Dentition” OR Dentures OR “Denture*”OR “Dental” OR “Oral” OR “Teeth”) OR TI (“Dentition” OR Dentures OR “Denture*”OR “Dental” OR “Oral” OR “Teeth”) OR AB (“Dentition” OR Dentures OR “Denture*”OR “Dental” OR “Oral” OR “Teeth”)

S14

S6 AND S13

S15 – Together – search with OR:

S6 OR S8 OR S10 OR S12 OR S14
Results: 600

Supplementary Table S3. Quality assessment of the included papers (n=8).

Author, year	Class	Quality rating (Negative, neutral or positive)	Was the research question clearly stated?	Was the selection of study subjects/ patients free from bias?	Were study groups comparable?	Was method of handling withdrawals described?	Was blinding used to prevent introduction of bias?	Were intervention/ therapeutic regimes/ exposure factor or procedure and any comparison(s) described in detail? Were intervening factors described?	Were outcomes clearly defined and the measurements valid and reliable?	Was the statistical analysis appropriate for the study design and type of outcome indicators?	Are conclusions supported by results with biases and limitations taken into consideration?	Is bias due to study's funding or sponsorship unlikely?
Brochier et al. 2018	D	Neutral	Y	U/C	U/C	N	U/C	Y	Y	Y	Y	Y
Feinberg et al. 1996	B	Neutral	Y	U/C	U/C	U/C	U/C	U/C	Y	U/C	Y	U/C
Hollaar et al. 2017	D	Neutral	Y	U/C	N/A	N/A	U/C	U/C	Y	Y	Y	Y
Langmore 1998	B	Neutral	Y	U/C	U/C	U/C	U/C	U/C	Y	U/C	U/C	U/C
Pu et al. 2017	D	Positive	Y	Y	Y	Y	U/C	U/C	Y	Y	Y	U/C
Rech et al. 2018 (a)	D	Neutral	Y	U/C	U/C	N	U/C	U/C	Y	Y	Y	Y
Rech et al. 2018 (b)	C	Neutral	Y	U/C	U/C	Y	U/C	U/C	Y	U/C	Y	Y
Sarabia-Cobo et al. 2016	B	Positive	Y	Y	N/A	N/A	N/A	Y	Y	Y	U/C	Y

Legend: Y – Yes, N – No, N/A – Not applicable, U/C – Unclear.
